# Pain assessment practice and associated factors among nurses working at adult care units in public hospitals in Wolaita Zone, Southern Ethiopia, 2021

**DOI:** 10.1186/s12912-022-00892-4

**Published:** 2022-05-13

**Authors:** Badeg Melile Mengesha, Fikre Moga Lencha, Lankamo Ena Digesa

**Affiliations:** 1 Tikur Ambesa Comprehensive Specialized Hospital, Addis Ababa, Ethiopia; 2 School of Nursing, College of Medicine and Health Sciences, Arba Minch University, Arba Minch, Ethiopia

**Keywords:** Nurses, Pain assessment, Practice, Ethiopia

## Abstract

**Background:**

Using standard pain assessment techniques is a cornerstone for effective pain management. Pain is not assessed in a standardized manner in numerous practice settings. The problem of applying pain assessment principles was found to be higher in low-income countries. Very limited evidence indicates the standard of pain assessment practice among nurses in Ethiopia. Therefore, the purpose of this study was to identify pain assessment practice and associated factors among nurses working at adult care units in public hospitals in the Wolaita Zone, Southern Ethiopia.

**Methods:**

A cross-sectional study was conducted among 290 nurses working at adult care units in public hospitals in Wolaita zone. Data were collected from February to March 2021. A structured self-administered questionnaire was used for data collection. Epi Data 4.6 was used to enter the data, and SPSS version 26 was used to analyze the data. A logistic regression model was used, and statistical significance was declared at P ≤ 0.05. An adjusted odds ratio with 95%CI was used to measure the degree of association.

**Results:**

A total of 267 nurses participated in the study, giving a response rate of 97.8%. Almost three-fourths (73.8%) of the study nurses reported that they assessed pain for their patients. Only 23.6% of the study nurses discussed pain assessment scores during a nurse-to-nurse report. Below, half (47.2%) of the study participants documented pain assessment scores. The proportion of nurses with good pain assessment practice was found to be 38.2%.

The odds of having good pain assessment practice among nurses who received training on pain management was two times higher than its counterpart. The nurses who perceived organizational support were twice more likely to have good pain assessment practice than their counterparts.

**Conclusion:**

Nurses’ pain assessment practice was found to be low. Moreover, a substantial proportion of the study nurses did not discuss pain assessment scores during a nurse-to-nurse report with low documentation practice. Continuous professional development through in-service training and education is crucial to the improvement of nurses’ pain assessment practice. Furthermore, ameliorating organizational support by means of a supportive working environment is suggested to the betterment of nurses’ assessment practice.

## Background

Pain is an unpleasant sensory and emotional experience associated with actual or potential tissue damage or described in terms of such damage [1, 2]. The evidence shows that pain is one of the main reasons patients seek medical care [3–6]. Untreated pain has an impact on patients' clinical and psychological well-being, as well as hastens their risk of mortality and has a slew of other negative repercussions, such as raising treatment expenses, lengthening hospital stays, and lowering their quality of life [2, 7–11].

Pain is regarded as the fifth vital sign [12–14] and as important as the other vital signs [15]. Assessment of pain is the first and most important step in pain management [4, 16–19]. It is notable that assessing pain decreases overtreatment and treatment-related adverse effects [16, 17, 20]. Pain assessment must be considered on admission, after a change in medical status, prior to, during, and after procedures [17]. A comprehensive assessment of pain requires subjective and objective data, self-reports, and assessment tools [17]. Actually, a patient’s self-report of pain is the most valid method of assessing pain in a patient who can communicate [6, 21, 22]. Moreover, patients' vital signs are part of a comprehensive pain assessment [16, 22–24]. Because the vital signs cannot distinguish pain from other types of suffering, they are utilized as a complement in pain assessment [22]. Additionally, during pain assessment, health care providers should also pay attention to patients’ behavior, such as facial expressions and gestures [25, 26]. Furthermore, assessment results must be documented and disseminated to all those involved in a patient's care [12, 17].

Nurses are in a unique position to support patients who are experiencing pain [4, 10, 27, 28]. Nurses play a vital role in providing pain assessment and management [16]. Besides, in order to effectively manage pain, a nurse must accurately measure the patient's experience of pain [8, 17, 18]. In addition, nurses should use a consistent and methodical approach to pain exploration [17]. Furthermore, nurses should consider pain assessment principles while using assessment techniques and instruments [25].

In many clinical contexts, pain is not assessed in a standardized manner [29]. Implementing the principles of pain assessment remains a challenge for many nurses [30, 31]. Several studies from developed countries pointed out that nurses’ pain assessments vary across regions, with some reports suggesting poor compliance among nurses in recording and reporting pain assessment findings [31–35]. On the other hand, problems of applying the standard of pain assessment were shown to be more prominent in low-income countries [36]. Moreover, African patients are less likely to verbalize their pain because expressing pain is seen as an act of weakness [37]. Thus, African regions suffer from a double burden of pain assessment from nurses’ and patients’ sides. Furthermore, similar results were reported from studies of African countries [15, 25, 38, 39], indicating poor adherence to the principles of pain assessment. Besides, the previous few and inconsistent studies in Ethiopia showed that pain assessment practice among nurses was from 24.4% to 55.9% [39–41].

Being a male nurse, having low work experience, a lower educational level, lack of training on pain management, lack of organizational support, and absence of pain management guidelines were associated with nurses’ poor pain assessment practice [39–42].

Previous studies in Ethiopia have mainly focused on pain management, other than pain assessment practice. Thus, there is very limited and inconsistent evidence on pain assessment practice among nurses in Ethiopia. Therefore, this study aimed to identify pain assessment practice and associated factors among nurses working at adult care units in the Wolaita Zone, Southern Ethiopia.

## Methods

### Study area and period

The study was conducted in Wolaita Zone in Southern Ethiopia. Currently, the Zone has six public hospitals, 67 health centers, and 342 health posts. The hospitals provide outpatient, emergency treatment, surgery, delivery, laboratory, and pharmacy services. The total nursing staff in hospitals was 596, of which 290 nurses were in adult care units. The study was conducted from February to March 2021 in public health facilities in Wolaita Zone, Southern Ethiopia.

### Study design

An institution-based cross-sectional study design was used.

### Study population

All nurses working in adult care units in public hospitals in the Wolaita Zone during the data collection period.

### Eligibility criteria

All nurses who were working in adult care units (medical ward, surgical ward, triage, emergency department, and intensive care units) participated in the study; nurses who were available during the data collection period and nurses working in the units for a minimum of six months were included.

### Sample size and determination

A single population proportion formula was used to calculate the sample size by using the following assumptions: The magnitude of good assessment practice was 57.1% from the previous study in Northern Ethiopia [41] with a margin of error of 5%, 95% confidence interval, and a 10% non-response rate. Finally, the required sample size was found to be 414. But only 290 nurses were working in adult care units in public hospitals during data collection. Therefore, all nurses working in six public hospitals were selected for the study.

### Sampling technique and procedure

All nurses working in six public hospitals were included in the study. A total enumeration was used because the calculated sample was greater than the total population of the study areas.

### Data collection tools and procedures

Data were collected using a self-administered questionnaire. The data collection tool was adopted from literature [39–41, 43]. The questionnaire addressed the following sections: socio-demographic characteristics and organizational factors, and practice assessing questions. Eight yes-or-no questions were used to identify nurses’ pain assessment practice. The response ‘’yes’’ indicated good practice, and the response ‘’no’’ was used to denote non-adherence to good practice. The data were collected by six trained nurses who had prior experience in data collection. Three supervisors oversaw the data collection process.

### Operational definition of terms

*Good practice*: nurses who reported an adherence of 70% or higher to the questions assessing pain assessment practice [40].

*Poor practice*: nurses who reported less than 70% adherence to the questions assessing pain assessment practice [40].

### Study variables

#### Dependent variable

Nurses’ pain assessment practice.

#### Independent variables

Age, marital status, educational level, work experience, working unit, pain management training, pain management guidelines availability, perceived organizational support, and reading pain-related references and journal articles.

### Data collection instrument quality assurance

The questionnaire was pre-tested on 5% of the total sample size outside of the hospitals before one week of data collection. The wording of the questionnaire and the time allocated to complete the questionnaire were modified based on the pre-test results.

### Data processing and analysis

Before entering data, the data were checked for completeness, then entered into Epi Data version 4.6, and analyzed with SPSS version 26. Descriptive statistics were done to see the overall distribution of study participants. The association between each independent variable and the dependent variable was checked by using binary logistic regression. All variables with a P ≤ 0.25 in the binary logistic regression analysis were taken to multiple logistic regression analysis to control the possible confounders. Adjusted odds ratio with 95% confidence interval and a *P*-value ≤ 0.05 was declared statistically significant.

### Ethical considerations

Ethical clearance was obtained from the Institutional Review Board of Addis Ababa University, College of Health Sciences, School of Nursing and Midwifery. An official letter was submitted to the Wolaita Zone Health Office, and approval was obtained from the organizations before data collection. All subjects provided written informed consent. And confidentiality of the information was maintained throughout the study.

## Results

### Socio-demographic characteristics and organizational factors

A total of 267 nurses participated in the study, giving a response rate of 97.8%. The mean age of the respondents was 28.7 (3.74 SD), with a minimum and maximum age of 22 and 42 years, respectively. The respondents had a mean of 5.4 years (2.9 SD) of work experience with a minimum and maximum of 1 and 16 years, respectively, as shown in Table 1 below**.**

**Table 1:** Socio-demographic characteristics and organizational factors of nurses working in adult care units at public hospitals, Wolaita Zone, Southern Ethiopia, 2021 (*n* = 267)

Variables	Category	Frequency	Percent
Sex	Male	142	53.2
	Female	125	46.8
Age(in years)	22–29	163	61
	≥ 30	104	39
Marital status	Never married	67	25.1
	Married	200	74.9
Educational level	Diploma	58	21.7
	Degree and above	209	78.3
Work experience	< 2 years	13	4.9
	2–5 years	141	52.8
	> 5 years	113	42.3
Working unit	Medical	73	27.34
	Surgical	140	52.43
	Emergency and ICU	54	20.22
Training on pain management	Yes	114	42.7
	No	153	57.3
Guideline of pain management	Yes	110	41.2
	No	157	58.8
Perceived organizational support	Yes	126	47.2
	No	141	52.8
Reading references and journal articles	Yes	59	22.1
	No	208	77.9

*ICU* Intensive Care Unit

### Nurses’ pain assessment practice

Almost three-fourths, 197(73.8%), of the study nurses reported that they assessed pain for their patients. Nearly two-thirds (76.4%) of the study participants did not discuss pain scores during a nurse-to-nurse report, as shown in Table 2 below**.**

**Table 2:** Pain assessment practice among nurses working in adult care units at public hospitals, Wolaita Zone, Southern Ethiopia, 2021 (*n* = 267)

Variables	Response	Frequency	Percent
Assess pain for their patients	Yes	197	73.8
	No	70	26.2
Use self-report of pain as a valid measure of pain if a patient is able to communicate	Yes	144	53.9
	No	123	46.1
Use pain assessment scales/tools	Yes	142	53.2
	No	125	46.8
Assess patients’ pain before and after a procedure	Yes	147	55.1
	No	120	44.9
Document pain assessment scores	Yes	126	47.2
	No	141	52.8
Discuss pain scores during a nurse-to-nurse report	Yes	63	23.6
	No	204	76.4
Use observation(patient’s behaviors and gestures) in pain assessment	Yes	120	44.9
	No	147	55.1
Use vital signs as extra indicators of the intensity of a patient’s pain/as a cue for pain assessment	Yes	134	50.2
	No	133	49.8

**Fig. 1:**
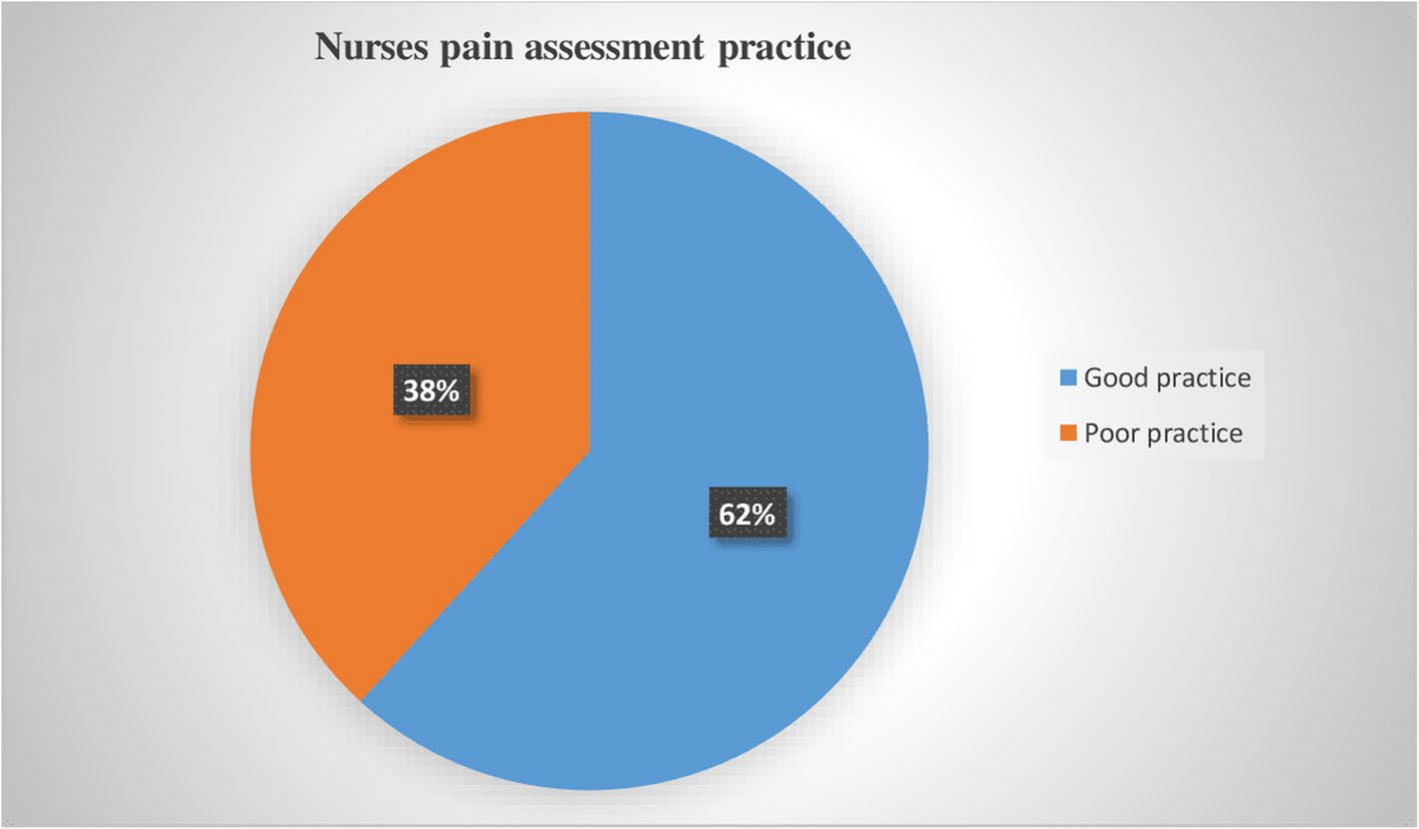
Pain assessment practice among nurses working at adult care units in public hospitals in Wolaita Zone, Southern Ethiopia, 2021 (*n* = 267)

Out of 267 nurses who participated in the study, 102 nurses had good pain assessment practice, as shown in Fig. 1.

### Multivariable analysis

The nurses who had training on pain management and perceived organizational support were twice as likely to have good pain assessment practice, as shown in Table 3 below.

**Table 3:** Multivariable regression analysis of nurses’ pain assessment practice among nurses working in adult care units at public hospitals, Wolaita Zone, Southern Ethiopia, 2021 (*n* = 267)

Variable	Category	Practice		COR(95% CI)	AOR(95%CI)	*P* value
		**Good Poor**				
Training on pain management	Yes	57	57	2.4(1.44–3.98)	2.2(1.26–3.87)	**0.005**
	No	45	108	1.00	1.00	
Guideline of pain management	Yes	67	90	1.59(0.95–2.66)	1.05(0.59–1.87)	0.864
	No	35	75	1.00	1.00	
Perceived organizational support	Yes	60	66	2.14(1.29–3.54)	1.9(1.2–3.23)	**0.017**
	No	42	99	1.00	1.00	
Marital status	Never married	30	37	1.44(0.822–2.53)	0.583(0.32–1.05)	0.073
	Married	72	128	1.00	1.00	

*AOR* Adjusted Odd Ration, *COR* Crude Odd Ratio

## Discussion

The majority of the nurses in this study, 73.8%, reported that they assessed pain for their patients. The result was higher than the findings of previous studies in Ethiopia [39, 41]. However, a higher magnitude was reported from other African countries [44, 45]. The discrepancy could be explained by the small sample size in the previous study in Ethiopia, and the study nurses from other African countries were from critical care units, so they may have had training and clinical skills in pain assessment.

The present study showed that 53.2% of nurses in the study used pain assessment tools. The finding was congruent with a study from Rwanda [45]. Nevertheless, the magnitude of this study was higher than several reports from Ethiopia and other African countries [15, 25, 39, 41]. These disparities could be attributable to the small sample size of the previous studies and differences in the instruments used for pain assessment.

In the current study, less than half (47.2%) of the study nurses documented pain assessment scores. The result was in accordance with findings reported by [31, 41, 45]. Contrary to the finding, studies from Africa and Asia [42–44] demonstrated lower results. The differences might be clarified by small sample sizes, and a non-probability purposive sampling technique was used in the previous studies.

According to this study, less than one-fourth (23.6%) of the study nurses discussed pain assessment scores during a nurse-to-nurse report. This suggests that the majority of nurses failed to report pain assessment scores. Several reports from other countries, including Ethiopia [15, 25, 40, 41] pointed out higher results than this study. It is by now generally accepted that discussing a patient’s pain assessment findings with a health care team is critical to advancing a patient's care.

In this study, the proportion of nurses with good pain assessment practice was found to be 38.2%. The result ties well with a previous study in Ethiopia [41]. Contrary to the findings, studies [33, 34, 39] and [40] reported a lower and higher magnitude, respectively. The possible reasons for the discrepancy could be due to some of the previous studies with smaller sample sizes and differences in a tool used for pain assessment practice. Moreover, the study [40] was conducted among nurses working in critical care units that might have better pain assessment skills.

The odds of having good pain assessment practice among nurses who received training on pain management was two times higher than its counterpart. This was consistent with what has been found in other African countries [25, 45]. However, having training on pain management was not associated with pain assessment practice, according to the study [40]. A similar conclusion was reached by previous studies wherein a lack of training on pain management leads to poor pain assessment practice [6, 46–49].

Finally, this study found a connection between nurses' pain assessment practice and perceived organizational support [AOR (1.9(1.2–3.23)]. This indicates that nurses who perceived organizational support were twice more likely to assess pain than those who did not. The finding was verified by previous studies [30, 43, 47]. Furthermore, providing organizational assistance allows nurses to improve their pain assessment skills [25, 50].

## Conclusion

Nurses’ pain assessment practice was found to be low. Moreover, most nurses did not discuss pain assessment scores during a nurse-to-nurse report. The problem of reporting pain is magnified by low documentation practice, which was below half. Continuous professional development through in-service training and education, and improving organizational support through good leadership are crucial to ameliorating nurses’ pain assessment practice. The management of the hospitals should continually support nurses through a supportive working environment and supervision. Future studies should address organizational and health service-related barriers in pain assessment. And reviewing records from patients’ charts to get a complete picture of pain assessment should be considered.

### Limitations of the study

This study might be subjected to self-report bias, and a record review from patients’ charts was not taken to get a complete picture of pain assessment practice. Another limitation of this study is that the study did not identify pain assessment tools used by nurses.

## Data Availability

The datasets used and/or analyzed during the current study are available from the corresponding author on reasonable request.
